# A successful surgical repair of intraoperative pneumothorax and the diffuse dissection of visceral pleura during liver transplantation surgery via trans-diaphragmatic approach

**DOI:** 10.1186/s40792-019-0568-y

**Published:** 2019-01-14

**Authors:** Kei Sakamoto, Akira Ogihara, Shota Mitsuboshi, Hideyuki Maeda, Takako Matsumoto, Tamami Isaka, Masahide Murasugi, Akiko Omori, Yoshihito Kotera, Hiroto Egawa, Masakazu Yamamoto, Masato Kanzaki

**Affiliations:** 1 Department of Thoracic Surgery, Tokyo Women’s Medical University, 8-1, Kawada-cho, Shinjuku-ku, Tokyo, 162-8666 Japan; 2 Department of Surgery, Institute of Gastroenterology, Tokyo Women’s Medical University, 8-1, Kawada-cho, Shinjuku-ku, Tokyo, 162-8666 Japan

**Keywords:** Pneumothorax, Trans-diaphragmatic approach, Liver transplantation

## Abstract

**Background:**

Pneumothorax during surgery under general anesthesia is a life-threatening situation for the patient because it can progress easily to the tension pneumothorax due to positive pressure ventilation unless appropriate treatments such as inserting a drainage tube in the thoracic cavity are initiated. The authors experienced a case of intraoperative pneumothorax and the diffuse dissection of visceral pleura during liver transplantation surgery, and achieved successful repair by a trans-diaphragmatic approach without changing patient’s body position.

**Case presentation:**

A 66-year-old male with multiple liver and renal cysts caused by autosomal dominant polycystic kidney disease (ADPKD) was admitted to the authors’ hospital for treating the infection of the liver cysts. The infection was unable to be controlled by conservative treatments. Therefore, the patient was planned to undergo living-donor liver transplantation. Intraoperatively, the liver was found to swell markedly and to firmly adhere to the right diaphragm. After the extraction of the liver, because the right diaphragm swelled markedly, pneumothorax was suspected. Chest tube was inserted immediately, and the small incision was made in the right diaphragm. Thoracoscopic observation revealed that (1) the visceral pleura of the bottom of the right lung widely expanded like a giant cyst due to the dissection from the lung parenchyma and (2) a large air leakage from a pin hole appeared in the dissected pleura. After the completion of the liver transplantation, the thoracoscopic leakage-closing operation was performed through the right diaphragm incision. Because the dissection of visceral pleura was too wide to perform plication or cystectomy by a stapler or sutures, the dissected pleura was opened, and absorbable fibrin sealant patches and fibrin glue were put or injected between the lung parenchyma and the pleura. Although, after being observed postoperatively, prolonged minor air leakage disappeared by a conservative drainage treatment, and the cyst on the bottom of the right lung disappeared on chest computed tomography (CT).

**Conclusions:**

Although intraoperative pneumothorax and broad dissection of visceral pleura during laparotomy is a complicated situation, the authors successfully repaired air leakage via a trans-diaphragmatic approach without changing the patient’s body position.

## Background

Pneumothorax during surgery under general anesthesia is a potentially critical situation for the patient, because it can progress easily to the tension pneumothorax due to positive pressure ventilation with an artificial respiration device unless appropriate treatments such as inserting a drainage tube in the thoracic cavity is initiated [1]. This report described (1) a case of intraoperative pneumothorax and the diffuse dissection of visceral pleura during liver transplantation surgery, and (2) the successful repair by a trans-diaphragmatic approach without changing the patient’s body position.

## Case presentation

A 66-year-old Japanese male was admitted to the authors’ hospital for fever and abdominal pain. He had a past history of multiple renal cysts, chronic renal failure on chronic hemodialysis for 2 years, and multiple liver cysts due to autosomal dominant polycystic kidney disease (ADPKD). He showed the relapse of infection of liver cysts and received longtime antibiotics therapies.

On admission, his vital signs were as follows: body temperature, 39.4 °C; heart rate, 101 beats per minutes (bpm); and respiratory rate, 20 bpm. Laboratory examination revealed a white blood cell (WBC) count of 7480 cells/mm^3^ with a high neutrophil count of 90.7%, and a C reactive protein of 18.08 mg/dl. He was diagnosed as systemic inflammatory response syndrome (SIRS). Diffusion-weighted magnetic resonance imaging (DW-MRI) showed an abnormal high intensity at the multiple liver cysts, indicating that polycystic infection was strongly suspected. Although antibiotic therapy with intravenous meropenem at a dose of 0.5 g/day was started, fever and inflammatory reactions were unable to improve. Therefore, percutaneous echo-guided drainage was introduced for the most accumulated liver cyst. Because of the improvements of clinical symptoms and laboratory findings, the drainage tube was removed at 19 days after drainage. However, the low-grade fever and inflammatory reactions recurred, and the discontinuation of antibiotic therapy was difficult. Because the most of the liver showed multilocular cystic changes, the control of infection was considered to be difficult by conservative treatments such as a local drainage or a partial liver resection, and the severe infection was expected to be repeated in the future.

As a fundamental treatment, a surgical approach was considered to be necessary, and a liver transplantation surgery was considered to be an indication for the disease. Therefore, he was planned to undergo living-donor liver transplantation with a right lobe graft from his wife. The graft was selected with considering a graft-to-recipient weight ratio of 0.88%, which is larger than the minimal recommended ratio of 0.8%.

**Fig. 1:**
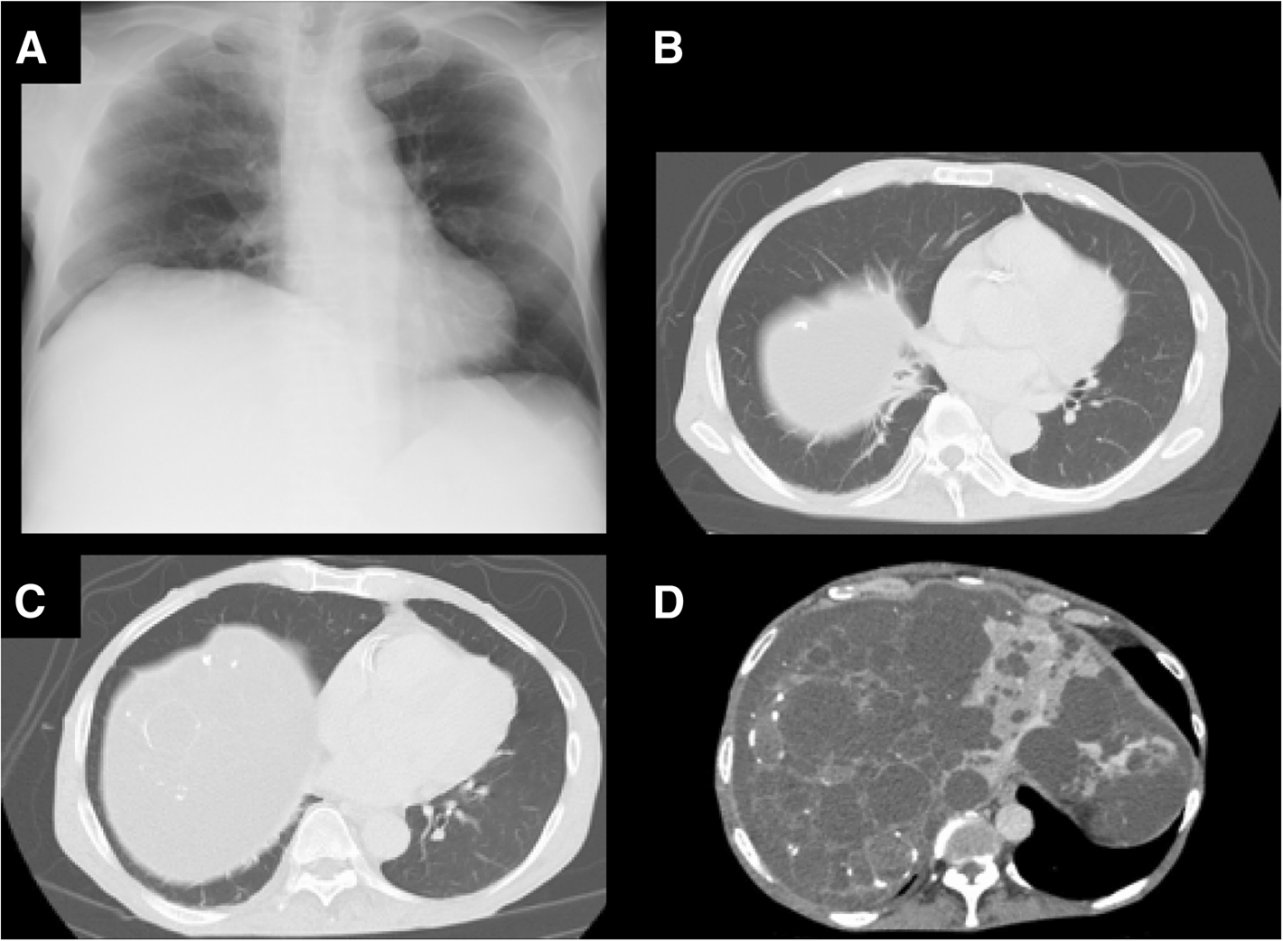
Preoperative chest X-ray and computed tomographic (CT) images of a 66-year-old man with multiple liver cysts. **a** Chest X-ray showed an elevation of right diaphragm. **b**–**d** CT showed hepatomegaly with multiple liver cysts and no cystic legions in the bottom of both sides of lung

Before the liver transplantation, he was 168.0 cm tall and weighed 68 kg. Physical examination revealed hepatomegaly extending below the level of the umbilicus. Laboratory data showed anemia (Hb 10.4 g/dL), and the elevation of C-reactive protein (2.61 mg/dL). Liver and renal profiles were as follows: total bilirubin, 0.8 mg/dL; albumin, 3.3 g/dL; aspartate aminotransferase (AST), 17 U/L; alanine aminotransferase (ALT), 10 U/L; international normalized ratio (INR), 1.3; creatinine, 5.09 mg/dL. His Child-Pugh score was 7 (grade B), and Model for End-Stage Liver Disease (MELD) score was 22. Arterial blood gas analysis showed that blood pH was 7.44, PaCO_2_ 32.9 mmHg, PaO_2_, 82.1 mmHg in room air, and alveolar-arterial oxygen tension gradient (A-aDO_2_) was increased to 26.8 mmHg. Chest X-ray revealed an elevation of right diaphragm. Computed tomography (CT) showed hepatomegaly with multiple liver cysts, multiple renal cysts, mild ascites, and no abnormal findings in the lung (Fig. 1). Respiratory function test showed the forced vital capacity (FVC) was mildly reduced down to 72% of predicted value, and the adjusted diffusing capacity of the lungs for carbon monoxide (DLco) by Cotes’ method was moderately reduced down to 57.8% of predicted value. Venous ultrasonography revealed lower extremity venous thrombosis, and the day before the operation, an inferior vena cava filter was inserted by a right internal-jugular-vein approach without any complications. At 114 days after admission, a living related liver transplantation was performed. After endotracheal intubation, two central venous catheters were inserted from left and right internal jugular veins before surgery without any complications.

General anesthesia was induced with propofol and remifentanil, and maintained with sevoflurane and remifentanil. The artificial ventilation was uneventful, and the airway pressure was kept below 30 cmH_2_O during the intraoperative period.

The abdominal cavity was reached through the upper abdominal reverse-T incision. The liver was found to swell markedly and to push the right diaphragm upward. Although the liver was firmly adhered to the right diaphragm, the adhesion was sharply dissected by electrocautery under appropriate traction. After the extraction of the liver, the right diaphragm was found to swell markedly and occupy the abdominal cavity. Although there was no significant change in the vital signs, the right pneumothorax was suspected, a thoracic drain was immediately inserted through the right tenth intercostal space, and continuous drainage was initiated. However, the right diaphragm distended by a massive air leak and prevented the operators from anastomosing the liver vessels and the bile duct. Therefore, a small relaxing incision was made in the right diaphragm for depressurizing the right thoracic cavity and making an adequate operative field to anastomose. Thoracoscopic observation was performed through the diaphragm incision. There was no adhesion in the right thoracic cavity, and the visceral pleura of the bottom of the right lung was widely expanded like a giant cyst due to dissection from the lung parenchyma, and a marked air leak was recognized from a pin hole in the dissected pleura. Since controlling the air leakage in the patient was considered difficult only by drainage, a leakage closure operation was decided to be performed after the completion of the liver transplantation. Following the transplantation procedure, the thoracoscopic leakage closure operation was performed for the patient in the supine position.

**Fig. 2:**
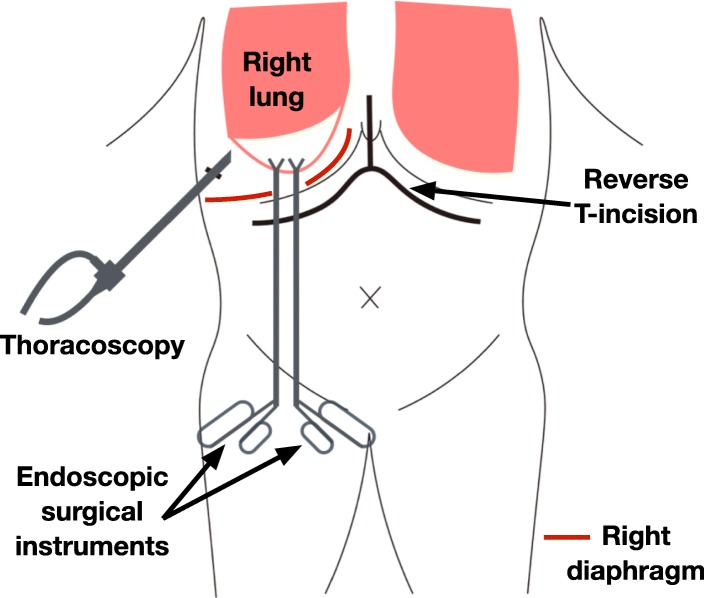
Schematic illustration of a trans-diaphragmatic approach. A thoracoscope was inserted through the right tenth intercostal space, and endoscopic surgical instruments were inserted through the small incision of right diaphragm

**Fig. 3:**
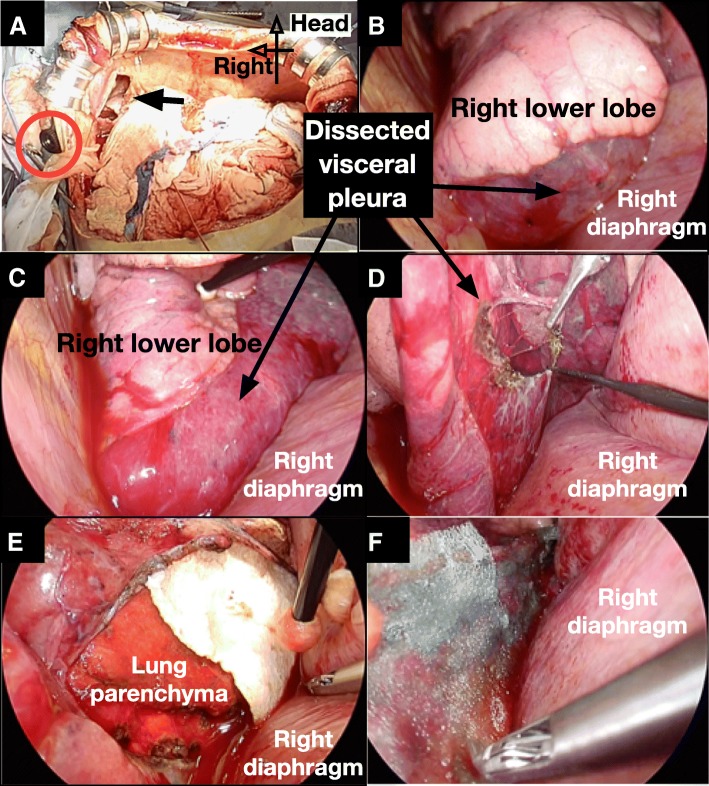
Surgical procedure. **a** Surgical view through the reverse-T incision. A small incision was made in the right diaphragm (Arrow). An 11.5-mm port was inserted in the right tenth intercostal space (Red circle). **b**, **c** The visceral pleura of the bottom of right lower lobe was widely dissected from lung parenchyma and presented like a giant cyst. **d**, **e** The dissected pleura was opened, and fibrin sealant patches were put on the lung parenchyma under the cyst. **f** Polyglycolic acid (PGA) sheets were also applied on the surface of the visceral pleura after closing the air leakage

**Fig. 4:**
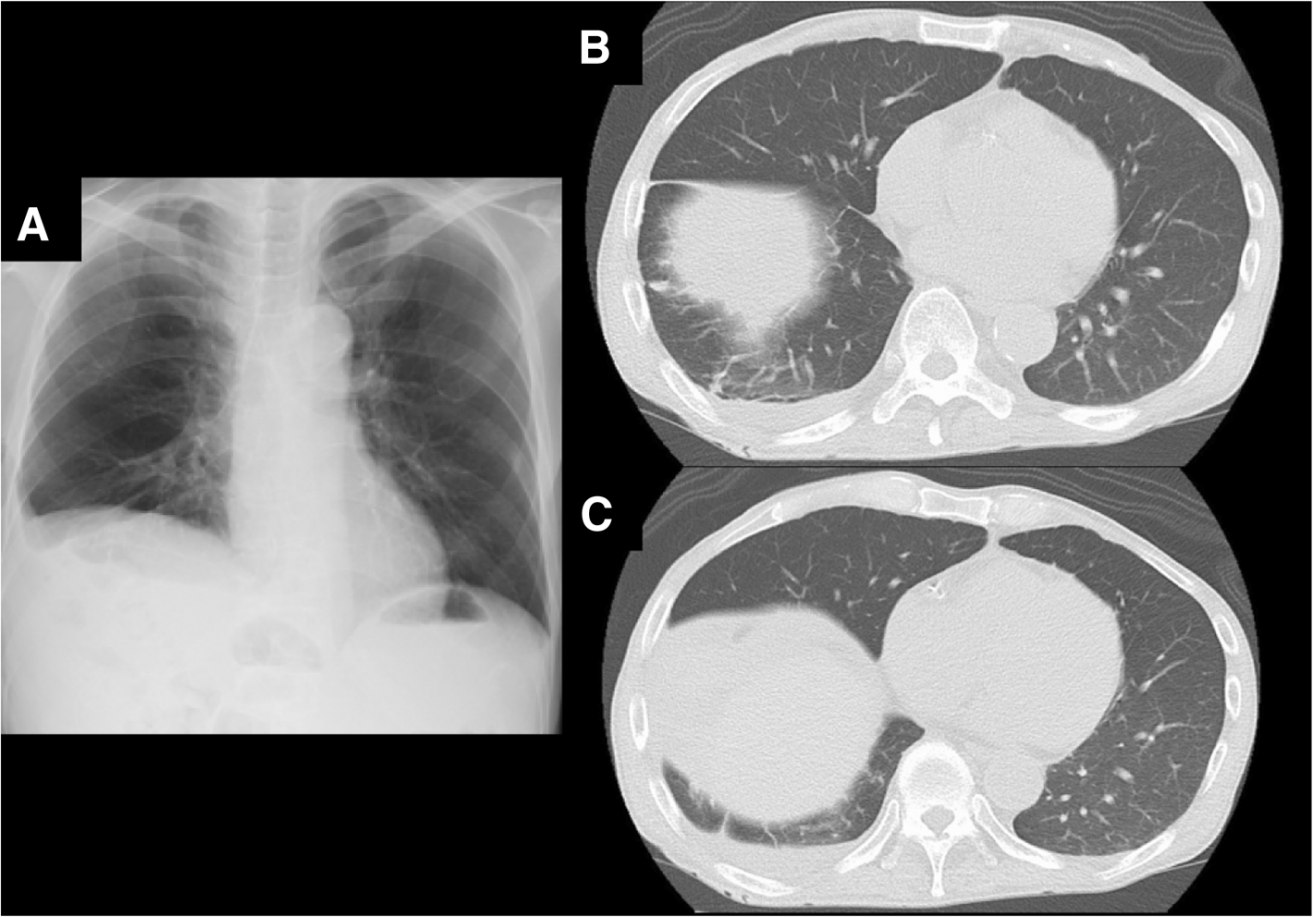
Postoperative chest X-ray and computed tomographic (CT) images taken at 3 months after surgery. **a** Chest X-ray showed the expansion of the right lung. **b**, **c** CT images showed that no cyst existed in the bottom of the right lung

During operation, a thoracoscope was inserted through the right tenth intercostal space, and endoscopic surgical instruments were inserted through the small diaphragm incision (Fig. 2). The expanded visceral pleura was incised by electrocautery to drain air and pooled blood in the cyst, and bleeding sites in the lung parenchyma were identified and controlled by electric coagulation. Because the lung parenchyma and the visceral pleura of the bottom of the lung was widely dissected and the base of the cyst was large, cystectomy was found to be difficult to be performed by a stapler or sutures. For controlling the air leakage, absorbable fibrin sealant patches (TachoSil®, Baxtor Healthcare, Deerfield, Illinois, USA) were patched on the lung parenchyma under dissected visceral pleura, and fibrin glue (Beriplast P®, CSL Behring, Tokyo, Japan) was injected into the dissected space. After no air pooling was confirmed under the visceral pleura with positive pressure ventilation, polyglycolic acid (PGA) sheets (Neoveil®, Gunze, Kyoto) were applied, and fibrin glue was sprayed on the visceral pleura surface (Fig. 3). Finally, a 21Fr silicone thoracic tube was inserted through the right 10th intercostal space and placed on the apex of right thoracic cavity, and the diaphragm and abdominal incision was closed. Although a postoperative minor air leakage was observed, chest X-ray showed the complete expansion of the right lung. The patient was treated with water seal drainage. After the air leakage disappeared, the drainage tube was removed at 27 days after surgery. The course after liver transplantation was also smooth, and he was discharged at 67 days after surgery. The cyst on the bottom of the right lung was confirmed to disappear on chest CT after discharge (Fig. 4).

## Discussions

Pneumothorax during surgery under general anesthesia is a serious life-threatening disease requiring immediate insertion of drainage tube in the thoracic cavity.

Respiratory complications during liver transplantation surgery are scarcely reported. Although there is a report on re-expansion lung edema due to the rapid expansion of lung during surgery [2], no report on pneumothorax was found.

Pneumothorax during liver transplantation surgery is considered as a rare situation, while there are some reported cases of intraoperative pneumothoraxes during general anesthesia [3–6]. From previous literatures, the speculated causes of the pneumothorax are as follows: an air leakage from the pre-existing emphysematous bullae, pneumoperitoneum for laparoscopy, erroneous puncture at the time of preoperative central venous catheters (CVCs) insertion, and barotrauma due to positive-pressure ventilation. However, the first three causes were unable to be applied to that of this case, because no emphysematous bulla was found on the preoperative CT image, no pneumoperitoneum was established, the air leakage site was at the bottom of the lung, suggesting that no relation was obviously found between preoperative CVCs insertion. On the other hand, a possibility that barotrauma due to positive pressure ventilation is the cause is unable to be denied. Reports show the risk of pneumothorax due to positive pressure ventilation, even though no excessive positive airway pressure is observed [7–11].

Also, in this case, the diffuse dissection of visceral pleura was observed. Although no reports describe the same symptom during operation under general anesthesia, some reports show subpleural air cysts as a complication of (1) pulmonary barotrauma during cardiopulmonary resuscitative treatments or (2) mechanical ventilation for adult respiratory distress syndrome [12–14]. Therefore, as a possible mechanism for the diffuse dissection of visceral pleura, barotrauma is speculated to induce partial pleural dissection, and the dissected space was spread broadly due to positive pressure ventilation.

Another possibility is that, in this case, under the chronically depression of the right lower lobe due to the uplift of the right diaphragm by markedly swollen liver, the extraction of the liver was speculated to induce a rapid expansion of the right lower lobe and damage the lung parenchyma, resulting in the dissection of visceral pleura.

Upon this surgery, two techniques were devised. First, the authors approached the air leakage point in the bottom of the right lung from the laparotomy through the right diaphragm incision. When performing surgical closure of pleural fistula, usually the lungs were manipulated through thoracoscopic ports on the chest walls in the decubitus position [﻿15﻿]. However, in this case, since (1) the patient’s body position was difficult to change during the liver transplantation, (2) small incision was already opened in the diaphragm, and (3) the air leakage point was the bottom of the lung, the authors decided to repair the air leakage by a trans-diaphragm approach in the supine position. Being slightly uncommon, the approach was thought to be a reasonable technique, because the approach was able to reach the operative field at the shortest distance and to allow surgical manipulation to be easy and to require no new incision.

Second, for closing the leakage, the dissected visceral pleura was incised widely, and the leakage was closed with several drops of sealant without resecting the lung cyst. For managing an air leakage from a lung cyst, the resection, ligation, or plication of the cyst is common [﻿16﻿–﻿18﻿]. However, in this case, because (1) no cyst was found in the bottom of the lung before the operation and (2) the broad dissection of visceral pleura was occurred during surgery, the resection or plication of the cyst was expected to be difficult. Therefore, the authors decided to open the cyst and patch the air leakage points with fibrin sealant patches and fibrin glue on the lung parenchyma, and PGA sheets and fibrin glue on the visceral pleura, and consequently the air leakage would be controlled. Several prospective studies report on the usefulness and safety of fibrin-sealant application to the lung parenchyma or PGA sheets to visceral pleura. For example, fibrin sealant is applied for the lung parenchyma of divided interlobar fissure during lobectomies, and treatments for postoperative air leakage during redo surgery for lung malignancies [﻿19﻿–﻿25﻿]. Moreover, similar to this case, giant bullas are reported to be cured only by covering the lung parenchyma with fibrin glue under the cyst wall without resecting the cyst [﻿26﻿]. These reports indicate that the choice of technique is considered reasonable.

Although the appearance of pneumothorax during open abdominal surgery was a relatively rare situation, it is a potentially critical situation, because it can easily develop to tension pneumothorax without appropriate diagnosis and treatment. Surgeons should be aware of the unexpected onset of pneumothorax during abdominal surgery, which could manipulate and pull the diaphragm. In this situation, the trans-diaphragmatic approach without change in body position used in this report would be hopefully one of effective options to reach the thoracic cavity.

## Conclusions

Surgical repair for pneumothorax and the broad dissection of visceral pleura during liver transplantation surgery was performed by a trans-diaphragmatic approach without changing the patient’s body position.

## Abbreviations

ADPKD: Autosomal dominant polycystic kidney disease
CT: Computed tomography
PGA: Polyglycolic acid
CVCs: Central venous catheters
FVC: Forced vital capacity
DLco: Diffusing capacity of the lungs for carbon monoxide
